# Improving Access and Mental Health for Youth Through Virtual Models of Care

**DOI:** 10.1007/978-3-030-51517-1_17

**Published:** 2020-05-31

**Authors:** Cheryl Forchuk, Sandra Fisman, Jeffrey P. Reiss, Kerry Collins, Julie Eichstedt, Abraham Rudnick, Wanrudee Isaranuwatchai, Jeffrey S. Hoch, Xianbin Wang, Daniel Lizotte, Shona Macpherson, Richard Booth

**Affiliations:** 8 Digital Research Centre of Sfax, Sfax, Tunisia; 9 Institut Mines-Télécom, CNRS, Paris, France; 10 Université de Sherbrooke, Sherbrooke, QC Canada; 11 Digital Research Centre of Sfax, Sfax, Tunisia; 12 University of Sfax, Sfax, Tunisia; 13 Lawson Health Research Institute, London, ON Canada; 14 Western University, London, ON Canada; 15 St. Joseph’s Health Care, London, ON Canada; 16 London Health Sciences Centre, London, ON Canada; 17 Department of Psychiatry and School of Occupational Therapy, Dalhousie University Halifax, Halifax, NS Canada; 18 Nova Scotia Operational Stress Injury Clinic, Nova Scotia Health Authority, Halifax, NS Canada; 19 St. Michael’s Hospital, Toronto, ON Canada; 20 Division of Health Policy and Management, Department of Public Health Sciences, University of California Davis, Davis, CA USA

**Keywords:** Smart technology, Youth, Mental health, eHealth

## Abstract

The overall objective of this research is to evaluate the use of a mobile health smartphone application (app) to improve the mental health of youth between the ages of 14–25 years, with symptoms of anxiety/depression. This project includes 115 youth who are accessing outpatient mental health services at one of three hospitals and two community agencies. The youth and care providers are using eHealth technology to enhance care. The technology uses mobile questionnaires to help promote self-assessment and track changes to support the plan of care. The technology also allows secure virtual treatment visits that youth can participate in through mobile devices. This longitudinal study uses participatory action research with mixed methods. The majority of participants identified themselves as Caucasian (66.9%). Expectedly, the demographics revealed that Anxiety Disorders and Mood Disorders were highly prevalent within the sample (71.9% and 67.5% respectively). Findings from the qualitative summary established that both staff and youth found the software and platform beneficial.

## Introduction

In Canada, the total cost of treatment, care and support services for mental health problems exceeds 42.3 billion Canadian dollars per year [1], with mental health services for young people being the second highest youth healthcare expenditure after injuries [2]. Although 70% of mental health problems develop during childhood and adolescence [3], only a quarter of the 10–20% of Canadian youth affected by mental illness will receive mental health services [4]. Suicide is the second leading cause of death among Canadian youth, accounting for 24% of the deaths among individuals aged 15–24 [4]. Research on the integrated use of information technologies has shown strong improvements in the accessibility, quality, and efficiency of health and mental healthcare services [5]. Mobile technologies, in particular, appear to be a promising avenue due to the ubiquitous and portable nature of mobile devices. Smart phones have been successfully used to complement the treatment of a wide range of illnesses such as schizophrenia [6], bipolar disorder [7], and social phobia [8].

This ongoing study is integrating a mobile technology solution into routine care for youth who have symptoms of anxiety and depression. This technology is expected to: 1) promote healthcare outcomes, community inclusion and quality of life; and 2) reduce healthcare system costs by preventing hospitalization and reducing the need for outpatient visits. This report focuses on baseline data and the initial set of focus group data with youth and their care providers.

## Materials and Methods

### Study Design

This participatory action research project utilized a pre-post, mixed methods design. This paper reports on the baseline data from interviews and the initial focus groups after the youth had been using the application for less than 3 months.

Semi-structured interviews are being conducted at baseline, 6, and 12 months respectively. Focus groups will be held with youth and separate groups with care providers. A standardized evaluation framework will be instituted to facilitate systematic effectiveness, economic, ethical, and policy analyses [9]. The primary outcome measure for effectiveness is the Community Integration Questionnaire – Revised.

### Participants

This two-year project will recruit 125 youth participants and have recruited 115 youth (ages 14–25) from the caseloads of 46 mental healthcare providers in London and Woodstock, Ontario, Canada who are receiving hospital-based or community agency-based outpatient care.

Additional inclusion criteria for participants to participate in the study include:

1. Must be on a caseload of a participating staff or care provider.
2. Able to understand English to the degree necessary to participate.
3. Have symptoms of anxiety or depression.
4. Youth must be 14–25 years old.

### Intervention

The study, called Youth Telemedicine and Patient-Reported Outcome Measurement (TELEPROM-Y), allows participants synchronous and asynchronous communication with their staff/care provider team through the Collaborative Health Record (CHR). The CHR integrates the workflow of the full spectrum of healthcare providers, while also having embedded patient engagement functionality. These functionalities include the ability to: book appointments online; track quality of health and health outcome scores using mobile devices; access tailored educational content pertaining to their mental health; and engage in both synchronous (e.g. video-conferencing) and asynchronous (e.g. secure messaging) virtual visits with their healthcare providers. Youth participants used a smartphone application (app) to connect to the CHR. The intervention is designed to facilitate better care and engagement between the patient and their care team.
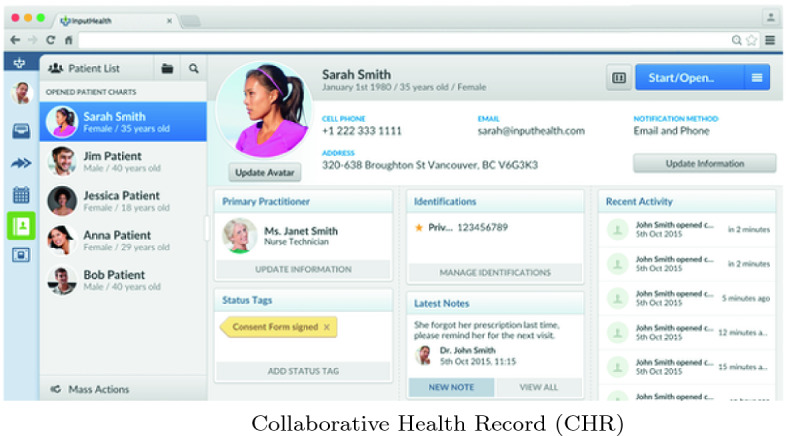
 
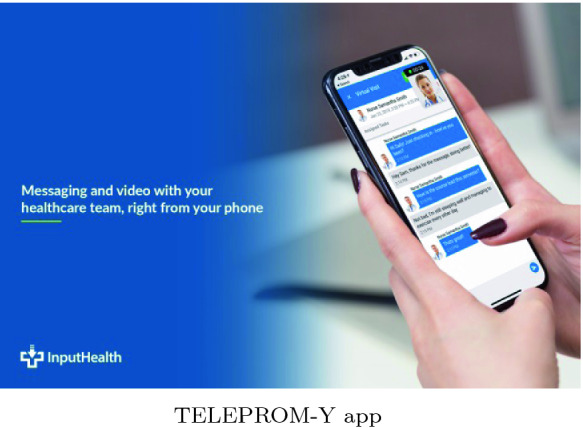


### Measures

Measures included a demographic questionnaire, the Community Integration Questionnaire [10], Lehman’s Quality of Life [11], EQ-5D, health and social services utilization, and Likert scales assessing perception of technology, a researcher-developed questionnaire that inquires about participants’ attitudes and opinions of the smartphone provided, provided data plan, and the CHR. Common qualitative items included feedback from participants on what they do and do not like about the technology, as well as suggestions for improvement on ethical principles such as autonomy, privacy and beneficence. A thematic analysis [12] using an ethnographic [13] method of analysis will be used to observe the broader social and cultural contexts surrounding individual experiences as well as the impact on staff/care providers and how the intervention influenced their practice.

## Results

### Data Analysis

**Fig. 1.**
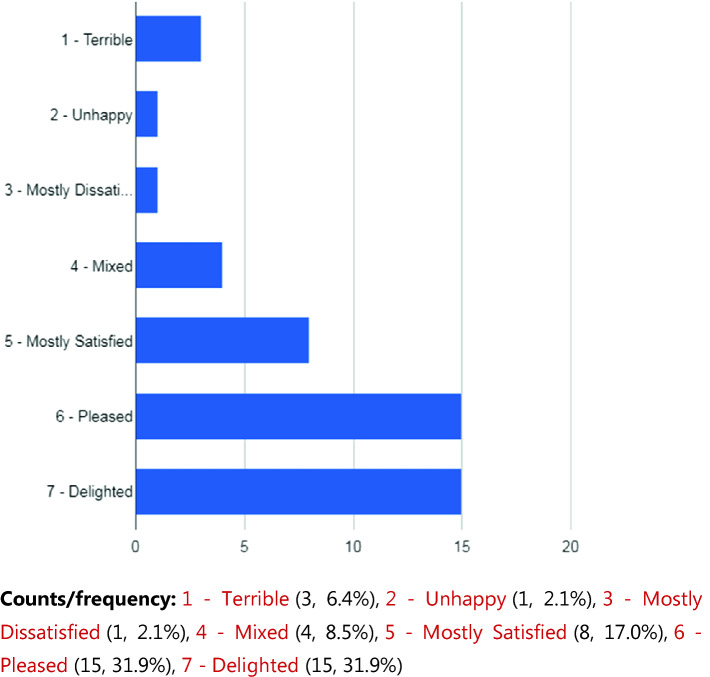
How do you feel about connecting with your care provider using your smartphone?

**Fig. 2.**
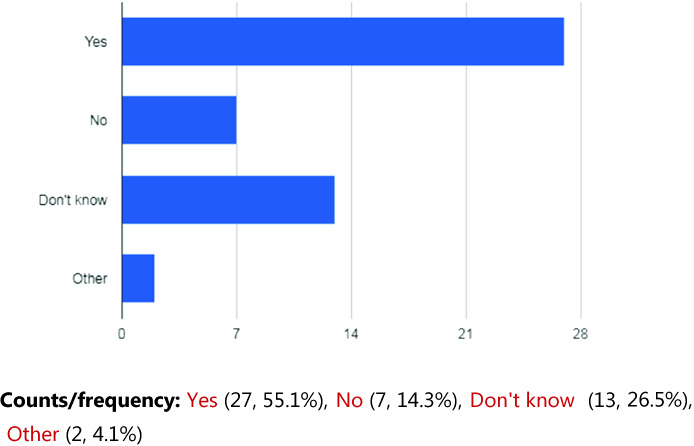
Has the use of the Smartphone and CHR for personal health information improved your healthcare?

At present, a total of 115 participants have been recruited into the study (see Table 1). There was a wide range of ages among the participants on enrollment from 14 to 25. The majority of participants identified themselves as Caucasian (66.9%) as shown in Table 1. Expectedly, the demographics revealed that Anxiety Disorders and Mood Disorders were highly prevalent within the sample (71.9% and 67.5% respectively). Of this sub-population of the sample who reported prior psychiatric admissions, the mean number of days since their most recent hospitalization was 68. The Perception of Smart Technology found that a lot of the youth participants were not using the CHR, nor had they even downloaded it. The individuals who had used it found that it improved their healthcare. See Figs. 1 and 2 (Table 2).

**Table 1.** Demographics (N = 115)

Age (mean)	19.57 yrs
*Sex*	
Female	63 (55.3%)
Male	51 (44.7%)
Other	1 (0.9%)
*Ethnicity*	
Caucasian	77 (66.9%)
Indigenous	12 (10.4%)
Black	6 (5.2%)
Asian	2 (1.7%)
Latin American	2 (1.7%)
Arab	1 (0.8%)
Other	5 (4.3%)
Missing	6 (5.2%)
*Psychiatric diagnosis*	
Anxiety disorder	82 (71.9%)
Mood disorder	77 (67.5%)
Disorder of childhood/adolescence	42 (36.8%)
Personality disorder	17 (14.9%)
Psychotic disorder	14 (12.3%)
Substance-related disorder	13 (11.4%)
Developmental handicap	7 (6.1%)
Other	24 (21.0%)
*Previous psychiatric hospitalization?*	
Yes	71 (62.3%)
No	43 (37.7%)
Missing	1 (0.9%)
*Age at first psychiatric hospitalization (mean) (n = 71)*	15
*Estimated total number of psychiatric hospitalizations (mean) (n = 70)*	5.9

**Table 2.** Community Integration (N = 115)

*Approximately how many times a month do you usually visit friends or relatives?*	
Never	14 (12.3%)
1–4 times	50 (43.9%)
5 or more	50 (43.9%)
Missing	1 (0.9%)
*When you participate in leisure activities do you usually do this alone or with others?*	
Mostly alone	25 (21.9%)
Mostly with friends who have mental health challenges	22 (19.3%)
Mostly with family members	11 (9.6%)
Mostly with friends who do not have mental health challenges	18 (15.8%)
With a combination of family and friends	38 (33.3%)
Missing	1 (0.9%)
*How often do you travel outside the home?*	
Almost every day	85 (74.6%)
Almost every week	25 (21.9%)
Seldom/never (less than once per week)	4 (3.5%)
Missing	1 (0.9%)
*How often do you write to people for social contact using the Internet (e.g., Facebook)?*	
Every day/most days	83 (72.2%)
Almost every week	21 (18.3%)
Seldom/never	11 (9.6%)
*How often do you make social contact with people by talking or text messaging using your phone?*	
Every day/most days	80 (70.2%)
Almost every week	14 (12.3%)
Seldom/never	20 (17.5%)
Missing	1 (0.9%)

### Initial Focus Groups

A staff focus group and 2 youth focus groups have been completed prior to 3 months of implementation.

Youth described the advantages of both the app as well as having a phone. For the application itself, youth identified increased communication with their care provider, primarily through the messaging function. They also appreciated having the availability of information on phone including safety plan, the ability to set up appointments and, reminders related to wellness plan, as well as medication prompts. They enjoyed using a paperless format for things such as completing forms on-line that were sent by the care provider. Examples of comments include:

*I liked doing the little survey thing. Cause like when I’m bored and or like on a bus or something and I think, need to stop focusing on people, like, probably not staring at me but staring at me. I’ll go on it. It gives me something to do so, and like it’s helpful to so. I’ll do like the surveys and questionnaires that pop*-*up …*

*Well I mean just yesterday, I was able talk to Dr.(Name), who scheduled an a, an appointment for Friday, which I found really helpful because I wouldn’t know how to contact her otherwise*

The youth also described areas for improvement. They described that they initially had to take time to figure out the functions. There were several complaints about the cumbersome log-in process. Although they identified that they understood the log-in privacy concerns they thought it could still be streamlined. Some examples of quotes include:

*At first it was confusing, but then figured it out. I don’t know, like I don’t know exactly how it’s confusing but like, it was like, it, it was new, …*

*Yeah randomly it signed me out the other day like it was signed in for like a good few weeks now and I was like thank you. And then it just randomly signed out and I was like damn. I remember my stuff but it’s just annoying how you would put it in every single time but like I know that’s for like confidentiality and like some people can’t go on it. But, kinda sucks sometimes.*

The other major theme related to the importance of having a phone. Many of the youth in the study were experiencing poverty and twenty (17.5%) reported being homeless. The phones themselves helped youth feel comfortable and connected to other people. They reported the advantage of having a phone at all. Some examples of quotes include:

*When I didn’t have a phone like I just hate going places and not knowing where I’m going, or like I don’t know, my anxiety’s really bad.*

*Then, the phones helped me to not only help myself, but to help others in like emergent situations… which I had to do a couple weeks ago and had I not gone to the study, like I wouldn’t have been able to help them so it’s helped in a lot of ways.*

Staff identified similar issues using the app in particular the ability to securely message their clients frequently. Specifically, staff identified the advantages were the ability to send questionnaires and to set appointments using the app. They discussed that sometimes youth felt more comfortable to first raise uncomfortable topics by phone/messaging and this resulted in strengthening the relationship between the youth and care provider and empowering the youth. Staff supporters also identified the importance of their clients having a phone at all.

Some examples of quotes include:

*Yeah I use messaging, especially one time um one of my youth didn’t have any minutes on their phone so we actually were a couple times messaging through the site*

*I think a good one for me is the mood Qnaire and the medication one. Um, because a lot of my youth, their mood fluctuates either in a single day* – *when I see them in the morning they could be doing amazing and then by the afternoon they’re just doing horrible. So, I can send them one of those and then kind of see like maybe what a trend is and figure out the trend and then that way I can better support them as well*

*So that they do make it to their appointments on time, and at their like right day. I’m able to text them ahead of time to remind them*

*So, they don’t want to like give away things in person like if its … going to cause them to cry. It … allows them to be more vulnerable*

*If anything it’s increased the relationships and made them like stronger and better*

For improvements they noted the app had a medical look to it that was not inviting to youth:

*It does, like I feel like if I’m a 23*-*year*-*old and I’m just looking and it’s this nice bright blue and white, but…it’s just reminds me of a doctor’s office and a lot of our youth may be triggered by doctor’s offices, or have had really bad experiences…*

They also noted that they do not spend much time on their computer so they really need a phone app themselves for the provider version.

## Discussion

The demographic findings from the baseline interviews were characterized by a wide age range (14–25 years) with a comorbidity of psychiatric illnesses. There were 62.3% of participants who stated that they had been admitted to a hospital for psychiatric reason. When asked the estimate total number of psychiatric admissions, the average number of hospitalizations among youth who did report a hospitalization was 5.9 times.

With regards to community integration the sample appeared to be socially isolated as only 43.9% visiting friends or relatives at least weekly. The majority of the sample used social media on most days.

Based on the preliminary findings, we report that the use of smart technology was successfully deployed to a range of youth with symptoms of depression or anxiety. Both staff and youth identified strengths of both having the application as well as having a phone. The ability to communicate more easily was noted as a particular strength that has the potential to improve access to care and support the therapeutic relationship. As previously noted in the literature, smartphones have been found to be successful in assisting individuals with bipolar disorder [6], and social phobia [7]. It is anticipated that the TELEPROM-Y project will be able to provide greater assistance to individuals with mental illness through enhanced access to resources and supports, as well as further opportunities for communication with care providers. Moreover, the use of smartphones may represent a more convenient approach to mental healthcare as opposed to in-person appointments or printed resources (i.e. brochures, information sheets) that are easily lost or damaged. As described in the preliminary focus groups of this study, some individuals may not wish to visit a healthcare provider’s office due to previous negative experiences. Some participants voiced that they like receiving care from the comfort of their own environment using the CHR app. This can also negate any potential missed appointments or concerns going unchecked, therefore providing early intervention and prevention.

From the perspective of care providers, the CHR also allows for greater monitoring of clients for early intervention and prevention. By using the app to complete the questionnaires in real time the care providers can be alerted to any potential mental healthcare crises that may have otherwise been unreported or unacknowledged. This approach could allow mental healthcare providers to see and communicate with more individuals in one day. By improving this connectivity with care providers, participants in this study can overcome barriers to care such as lack of money or transportation to attend appointments, and difficulty accessing much-needed services. In Canada alone, 64% of street-involved youth have reported difficulty accessing services [14].

A challenge for this project was encouraging hospital health care providers to adopt this technological approach. Although a number of hospital care providers embraced the technology and efficiency of the software available, others were skeptical. Due to the personal nature of the data being collected, some were concerned about the participants’ privacy. Through the CHR is fully compliant with jurisdictional standards of practice, professional standards and guidelines, some care providers still did not want to participate in the study, so our team recruited from community agencies.

Another unanticipated expense was the amount of smartphones and data plans needed. In our proposal we anticipated purchasing 40 smartphones and 40 data plans but since a lot of the youth participants are living in poverty and/or homeless, an additional 55 had to be provided with a smartphone and 73 with a data plan to participate.

This study was limited by not being controlled other than by the pre/post-intervention design; future research could benefit from a comparison with a similar cohort of participants who do not receive the intervention during the study period, perhaps as part of a waiting list that could later receive the intervention.

## Conclusion

The implications of this study could be far-reaching. This intervention may provide a more efficient approach that enhances connectivity with care services. Further, this intervention could represent a more efficient approach to mental healthcare by providing participants with greater opportunities to seek additional support and resources. We further anticipate that these extra supports will result in greater community integration among participants, which in turn could improve quality of life.
